# Recurrence of depressive disorders after interferon-induced depression

**DOI:** 10.1038/tp.2016.274

**Published:** 2017-02-07

**Authors:** W-C Chiu, Y-P Su, K-P Su, P-C Chen

**Affiliations:** 1 Department of Psychiatry, Cathay General Hospital, Taipei, Taiwan; 2 School of Medicine, Fu Jen Catholic University, Taipei, Taiwan; 3 Graduate Institute of Neural and Cognitive Sciences, China Medical University, Taichung, Taiwan; 4 Department of Psychiatry and Mind-Body Interface Laboratory, China Medical University Hospital, Taichung, Taiwan; 5 Institute of Occupational Medicine and Industrial Hygiene, National Taiwan University College of Public Health, Taipei, Taiwan; 6 Department of Environmental and Occupational Medicine, National Taiwan University College of Medicine and Hospital, Taipei, Taiwan; 7 Department of Public Health, National Taiwan University College of Public Health, Taipei, Taiwan

## Abstract

Interferon alpha (IFN-α)-treated patients commonly develop depression during the therapy period. Although most IFN-α-induced depressive disorders achieve remission after IFN-α therapy, no studies have examined the long-term mood effects of IFN-α treatment. We conducted a 12-year population-based cohort study of hepatitis C virus (HCV)-infected patients who were older than 20 years and had received IFN-α therapy. The sample was obtained from the Taiwan National Health Insurance Research Database. The cohort included patients with and without IFN-α-induced depression, matched randomly by age, sex and depression history, at a ratio of 1:10. The follow-up started after the last administration of IFN-α and was designed to determine the incidence of recurrent depressive disorder after IFN-α therapy. A total of 156 subjects were identified as having IFN-α-induced depression and achieving full remission after IFN-α therapy. The overall incidence of recurrent depressive disorders among patients with and without IFN-α-induced depression was 56.8 (95% confidence interval (CI), 42.4–76.1) and 4.1 (95% CI, 2.9–5.8) cases, respectively, per 100 000 person-years, *P*<0.001. The adjusted hazard ratios for recurrent depressive disorder were 13.5 (95% CI, 9.9–18.3) in the IFN-α-treated cohort and 22.2 (95% CI, 11.2–44.2) in the matched cohort for IFN-α-induced depression patients after adjusting for age, sex, income, urbanization and comorbid diseases. IFN-α-induced depression was associated with a high risk of recurrent depression. It was not a transient disease and might be considered an episode of depressive disorder. Continuation therapy might be considered, and further research is needed.

## Introduction

Major depressive disorder is a highly recurrent illness,^1^ and its recurrence is an important reason for its burden.^2^ Population studies have found a⩾40–75% lifetime recurrence of major depressive disorder among patients who have recovered from their first depressive episode.^3, 4, 5^ Although remitted major depressive disorder patients have been studied for a number of years,^6^ the understanding of the mechanisms underlying its recurrence remains limited because most studies investigate major depressive disorder during the acute phase.^7^

Interferon alpha (IFN-α)-induced depression is a common and severe psychiatric disorder in IFN-α therapy for hepatitis C virus (HCV)-infected patients. Therapeutic administration of IFN-α leads to depression in up to 50% of patients, and up to 30% of patients develop IFN-α-induced depression (a major depressive episode according to Diagnostic and Statistical Manual of Mental Disorders, 4th edn diagnostic criteria) within the first 3 months.^8, 9, 10^ In addition, the clinical benefit of IFN-α is compromised by its common and severe neuropsychiatric adverse effects. For example, almost all patients experience acute sickness, including symptoms of fatigue, malaise, myalgia, arthralgia, anorexia, apathy and cognitive impairment.^11, 12, 13^ IFN-α administration to humans replicates multiple pathologies central to depression, thereby providing support for the notion that endogenous cytokines that mediate innate immune responses can contribute to the state of depression.^14^

The risk of recurrence after IFN-α-induced depression remains unknown. In a previous prospective study, the incidence of IFN-α-induced depression in patients who achieved remission of depressive episodes by the end of IFN-α therapy was 59.1%, whereas the other 40.9% achieved remission within 12 weeks.^15^ However, some studies have reported the recurrence of depression and suicidal thoughts even 6 months after the end of antiviral treatment.^16^ Although most IFN-α-induced depression will achieve remission after IFN-α therapy, no studies have examined the long-term mood effects of IFN-α.

To investigate the long-term risk of the recurrence of clinical depression, we conducted a 12-year population-based cohort study using reimbursement claims from Taiwan's National Health Insurance Research Database to determine the incidence of recurrent depressive disorder after treatment with IFN-α in HCV patients.

## Materials and methods

### Data sources

The cohort sample was obtained from the research database of the Taiwanese National Health Insurance (NHI). The NHI program, implemented on 1 March 1995, provides compulsory universal health insurance that covers all forms of health-care services for 98% of the island's population. In cooperation with the Bureau of NHI, the National Health Research Institute of Taiwan randomly sampled a representative database of all NHI enrollees using a systematic sampling method for research purposes; the result of this sampling is known as the Longitudinal Health Insurance Database. There were no statistically significant differences in age, gender or health-care costs between the sample group and all enrollees, as reported by the National Health Research Institute. We utilized databases in the sample cohort, which included information on patient characteristics, including sex, date of birth, dates of visits and up to five visit diagnoses (by International Classification of Diseases, Ninth Revision (ICD-9) classification). These databases have previously been used for epidemiological research, and the information on prescription use, diagnoses and hospitalizations is of high quality.^17, 18^

Following strict confidentiality guidelines in accordance with personal electronic data protection regulations, the National Health Research Institute of Taiwan maintains an anonymous database of NHI reimbursement data suitable for research.^19^

### Identification of study sample

#### HCV subjects

We conducted a population-based cohort study that included patients older than 20 years who had a first-time diagnosis of HCV infection (ICD-9 codes 070.7, 070.41, 070.44, 070.51, 070.54, V02.62) without hepatitis B virus (HBV) infection (ICD-9 codes 070.2, 070.3, V02.61) or HIV infection (ICD-9 code 042) between 1 January 1997 and 31 December 2010. Because liver diseases are an important health problem in Taiwan, the government pays particular attention to this health issue and has devised guidelines for its diagnosis.^20^

#### IFN-α-exposed HCV subjects

IFN-α therapy in the HCV cohort was defined as any exposure to IFN-α therapy (ATC codes L03AB04, L03AB05, L03AB09, L03AB10 and L03AB11). Subjects who had any diagnosis of depressive disorders (ICD-9 codes 296.2, 296.3, 300.4 and 311) or had received any antidepressant therapy within the year before the first treatment of IFN-α were excluded. Because IFN-α therapy could be re-prescribed in the recurrent-HCV patients, any IFN-α therapy disrupted for 180 days was identified as another course of IFN-α therapy, and the time of follow-up was ended before the first day of the next IFN-α therapy course.

#### Subjects with IFN-α-induced depression

Our study included IFN-α-exposed HCV subjects who had at least three outpatient visits recorded or one inpatient incident reported with the admission diagnosis (ICD-9 codes 296.2, 296.3, 300.4 and 311) between the first day of IFN-α therapy and 30 days after the last prescription of IFN-α.^21^ Subjects with IFN-α-induced depression in full remission were defined as those who had no diagnosis of depressive disorders or and had not received any antidepressant therapy for at least 6 months.

#### IFN-α-treated HCV subjects without depressive disorder

These subjects were IFN-α-exposed HCV subjects who did not meet the definition of IFN-α-induced depression.

### Study design

#### IFN-α-treated cohort

All IFN-α-exposed HCV subjects were enrolled in our cohort study. Participants who had any depressive disorder diagnosis or had received antidepressant treatment within 1 year before IFN-α therapy were excluded. Patients with IFN-α-induced depression who did not achieve full remission were excluded.

#### Matched cohort

Because the psychiatric characteristics were different between the participants with and without IFN-α-induced depression, we further created a matched cohort to investigate the risk of recurrent depression. HCV patients with and without IFN-α-induced depression during IFN-α therapy were matched randomly by age, sex and depression history at a ratio of 1:10 (with IFN-α-induced depression versus without IFN-α-induced depression).

**Figure 1:**
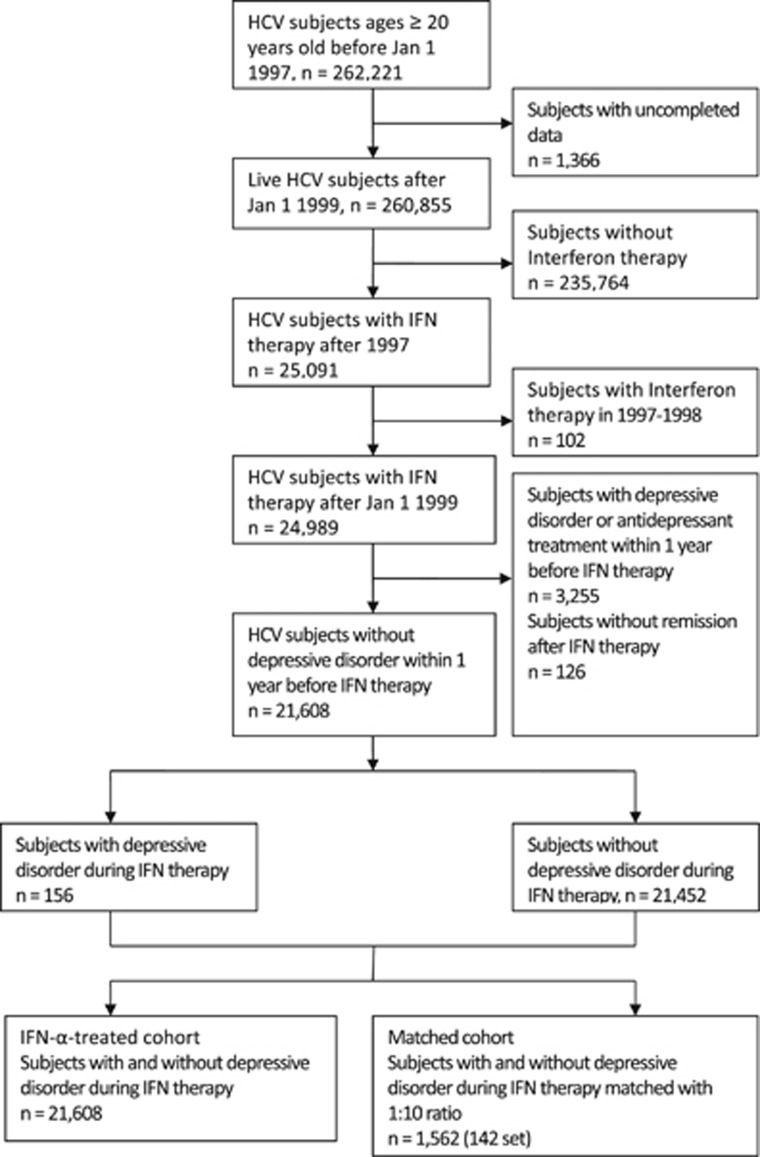
Flow of the Study Subjects Enrollment. We conducted a population-based cohort study from the Longitudinal Health Insurance Database of Taiwan, in which all patients older than 20 years who had a first-time diagnosis of hepatitis C virus (HCV) infection (International Classification of Diseases, Ninth Revision (ICD-9) codes 070.7, 070.41, 070.44, 070.51, 070.54 and V02.62) and received interferon (IFN) therapy between 1 January 1997 and 31 December 2010 formed the study cohort. The IFN-α-induced depression cohort comprised people who had the diagnosis of depressive disorder (ICD-9 codes 296.2, 296.3, 300.4 and 311) during the period between IFN-α therapy and the 30th day after the last IFN-α therapy. Patients with previous histories of depression diagnosis and antidepressant therapy before 1 year of IFN-α therapy were excluded when enrolling both cohorts. To ensure the remission of IFN-α-induced depression, the patients should have at least 6 months of free interval (without any diagnosis of depressive disorders or receiving any antidepressant therapy) after IFN-α induced depression. (Right bottom box) IFN-α-treated cohort: (*n*=21 608). (Left bottom box) Matched cohort: matching was performed on the characteristics of sex, age, history of depression with 1:10 ratio of the IFN-α-induced depression and without IFN-α-induced depression (*n*=1562 (142 pairs)).

The follow-up of both the IFN-α-treated and -matched cohort started on the day of the full remission of IFN-α-induced depression or the 31st day after the last prescription of IFN-α. All incident depressive cases (until censoring or matched censoring due to death, lack of follow-up or other factors) before the end of 2010 were included to explore whether the IFN-α-induced depression cohort had an increased risk of developing recurrent depressive disorder (Figure 1).

### Outcome

All diagnosed recurrent depressive subjects had at least three visits recorded in the outpatient records within 1 year or one incident recorded with the admission diagnosis (ICD-9 codes 296.2, 296.3, 300.4 and 311) during the follow-up.^22^

### Potential confounding factors

We systematically identified any risk factors for depression as potential confounding factors, defined as the following characteristics recorded before the date of IFN-α therapy: age, sex, income, urbanization, comorbidity and drug use (antidepressants and zolpidem). Urbanization levels in Taiwan were divided into four strata according to Taiwan National Health Research Institute publications, with level 1 referring to the 'most urbanized' and level 4 referring to the 'least urbanized' communities. Comorbid diseases included hypertension (ICD-9 code 401), diabetes (ICD-9 code 250), hypercholesterolemia (ICD-9 code 272), chronic pulmonary obstructive disease (ICD-9 codes 491, 492), liver cirrhosis (ICD-9 codes 571.2, 571.5, 571.6, 572.2, 572.3, 572.4, 572.8 and 573.0), coronary heart disease (ICD-9 codes 414, 429.2), thyroid disease (ICD-9 codes 240-246) and anemia (ICD-9 codes 280–285).

Antidepressant use in the cohort included tricyclic antidepressants, selective serotonin re-uptake inhibitors, monoamine oxidase inhibitors, serotonin–norepinephrine reuptake inhibitors, trazodone, bupropion and mirtazapine.

### Statistical analysis

We compared the distribution of demographic factors and the proportions of comorbidities between the IFN-α-induced depression and the non-IFN-α-induced depression groups in the IFN-α-treated and -matched models. The crude incidence rates of recurrent depression were calculated from the follow-up period until the end of 2010. We used the Kaplan–Meier method to estimate recurrent depression cumulative incidences. A Cox proportional hazard model was used to compute the hazard ratios (HRs), and the accompanying 95% confidence intervals (CIs) were used after adjusting for the variables mentioned. Finally, a log-rank test was performed to examine the differences in the risk for depressive disorder between the populations with and without IFN-α-induced depressive disorder. A two-tailed *P*-value of 0.05 was considered significant.

To examine potential effect modifiers, we conducted analyses stratified by age, sex and groups with and without diabetes, hypertension, hypercholesterolemia, chronic obstructive pulmonary disease, liver cirrhosis, anxiety disorder, use of zolpidem and histories of depression and antidepressant therapy in the cohort. These sensitivity analyses evaluated the differences and consistencies among age, sex and comorbidities. All analyses were conducted using the SAS statistical software (version 9.4; SAS Institute, Cary, NC, USA).

## Results

A total of 24 989 HCV-infected patients received IFN-α therapy from 1999 to 2010. There were 3255 patients who were excluded because of a depressive disorder diagnosis or the use of antidepressant therapies during the year preceding IFN-α therapy, and most of them (95.4%) received antidepressant treatments or therapies for depressive disorder during the IFN-α therapy period. There were 282 patients who developed depressive disorders during the IFN-α therapy period; of these patients, 126 (45.7%) did not achieve full remission and were excluded. The 156 patients who achieved full remission formed the IFN-α-induced depression cohort, and the other 21 452 HCV subjects formed the non-IFN-α-induced depression cohort (Figure 1). Table 1 shows the distribution of the subjects' demographic characteristics and medical conditions. The mean age was 54.4±10.4 years, and 44.2% of subjects were female. There were no significant differences in age, sex, income, urbanization or comorbidities between the groups with and without IFN-α-induced depression. The proportions of psychiatric conditions (anxiety disorder, zolpidem use and history of depression) were significantly higher in the IFN-α-induced depression group than in the IFN-α-treated cohort. After matching by age, gender and history of depression, only the zolpidem treatment was significantly higher in the IFN-α-induced depression group. Patients with IFN-α-induced depression also received a higher IFN-α dosage than subjects without IFN-α-induced depression.

The HRs of recurrent depression were significantly higher in IFN-α-induced depression patients. The crude HRs for recurrent depressive disorder were 14.4 (95% CI, 10.6–19.4) and 19.0 (95% CI, 10.6–34.3) for IFN-α-induced depression patients in the IFN-α-treated and matched cohorts, respectively. The adjusted HR for recurrent depressive disorder was 13.5 (95% CI, 9.9–18.3) for IFN-α-induced depression patients after adjusting for age, sex, income, urbanization and comorbidities. In the matched cohort, the adjusted HR for recurrent depressive disorders was 22.2 (95% CI, 11.2–44.2).

Fifty-eight (37.2%) IFN-α-induced depression incidents occurred in the first 45 days of IFN-α therapy, and one-third of patients with IFN-α-induced depression ended IFN-α therapy within the next two dosages. One hundred and twelve (71.8%) patients with IFN-α-induced depression received antidepressant therapy. We analyzed the risks associated with IFN-α-induced depression, including depression histories, early or delayed onset and termination soon after IFN-α-induced depression (Table 2). All results indicated a significantly higher risk of recurrent depression in the IFN-α-treated and matched cohorts.

### Sensitivity analysis

Being aged above 60 years, being male, having a anxiety disorder, having a history of depressive disorder and using zolpidem presented statistically significant associations with recurrent depressive disorder risk, as revealed by traditional multivariable Cox regression models (Table 3). This adjustment had little effect on the estimates from different models of associations between IFN-α-induced depression and recurrent depression. Table 4 also shows that the effects of IFN-α-induced depression remained significant against recurrent depression in the subgroup and sensitivity analysis.

## Discussion

To the best of our knowledge, this was the first nationwide study to examine the association between recurrent depressive disorder and IFN-α-induced depression in an HCV-infected population. The primary finding of our 12-year, population-based cohort study suggested that patients with a history of IFN-α-induced depression had a significantly higher risk for recurrent depression even without IFN-α exposure. Our secondary finding was that the recurrence of depressive episodes after IFN-α-induced depression was not associated with several clinical factors, including a history of depression and late-onset IFN-α-induced depression or antidepressant therapy. Regardless of patient characteristics, the risk of IFN-α-induced depression was high.

IFN-α-induced depression might predict later depressive episodes. In our study, only patients with remitted IFN-α-induced depression were included. In a previous prospective study, most IFN-α-induced depression achieved remission within 12 weeks after the end of the IFN therapy.^15^ Our study included 282 patients who developed depressive disorders during the IFN-α therapy period, after the exclusion of 3255 patients with depressive disorders and antidepressant use within the year preceding IFN therapy. IFN-α-induced depression was not always remitted after the completion of IFN therapy. Only 156 patients achieved at least 6 months of full remission, and the other 126 (45.7%) patients still received continuous treatment for an average of 2.3 years. Although our patients with IFN-α-induced depression who achieved full remission might have had a better prognosis, the risk of recurrent depressive disorder was still significantly high.

There was no difference in recurrence between early- and late-onset IFN-α-induced depression. In previous studies, patients who developed symptoms early in the course of IFN-α had predominantly neurovegetative and somatic symptoms and were less responsive to antidepressant treatment.^23^ Almost half of IFN-α-induced depression occurred in the first 2 months of our study, and there was no difference in the recurrence risk among the IFN-α-induced depression onset categories of less than 45 days, 46–90 days and more than 90 days. Although more patients experienced IFN-α-induced depression soon after IFN-α therapy, the onset time was not associated with the risk of recurrent depressive disorder.

Antidepressant therapy during IFN-α therapy might not decrease the risk of successive depressive episodes. Approximately 70% of IFN-α-induced depression patients received antidepressant therapy, and all of them achieved full remission and were free of antidepressants for at least 6 months in our study. Previous research revealed that the use of antidepressants to treat IFN-α-induced depression was followed by a significant reduction in the depression score.^24^ Although studies have shown that antidepressants do ameliorate IFN-α-induced depression and have a good response rate,^25^ the antidepressant therapy did not decrease the risk of recurrence in our study.

The biological mechanisms for the high recurrent risk after IFN-α-induced depression have never been investigated. However, it seems relevant to apply the concept of ‘kindling theory,' which indicates a pattern of reducing environmental thresholds with each successive depressive episode.^26^ Clinically significant negative mood following acute tryptophan depletion only in recovered depressed individuals was found to be explained by the kindling effect.^27^ Tryptophan was depleted in serotonin metabolism by the enzyme indoleamine 2,3-dioxygenase (IDO), which was induced by proinflammatory cytokines such as interleukin-1β, IFN-α, IFN-γ and tumor necrosis factor-α.^28^ IFN treatment of HCV-infected patients resulted in a decrease in plasma tryptophan and an increase in plasma kynurenine.^29^

In addition to tryptophan depletion, some underlying vulnerabilities for recurrence were associated with IFN-α-induced depression. A previous study showed an association between IDO gene variant (rs9657182) and significant depressive symptoms during chronic exposure to IFN-α, suggesting that IDO may have an important role in cytokine-induced behavioral changes.^30^ Patients carrying the G allele of the *HTR1A* gene, which might alter transcription in serotonergic and non-serotonergic neurons and confers a higher susceptibility to depression and suicide,^31^ were at a higher risk of depression during antiviral treatment.^32^ Moreover, some genetic variants that had relationships with depression, such as being homozygous for the A allele of the GCR1 polymorphism, the genotype GG of the brain-derived neurotrophic factor polymorphism and phospholipase A2 BanI polymorphism, were associated with IFN-α-induced depression.^33^ Other theory was also mechanistically insightful in the development of recurrent depression in IFN-α-induced depression patients by hypothalamic–pituitary–adrenal (HPA) axis dysregulation. The HPA axis response to the acute administration of IFN-α^34^ and chronic exposure to innate immune cytokines may contribute to the altered diurnal HPA axis activity and behavior.^35^ The HPA axis dysregulation, such as lower cortisol levels^36^ and diurnal profiles of dehydroepiandrosterone-sulphate and cortisol/dehydroepiandrosterone-sulphate ratio,^37^ may predict future recurrence and suggest an endophenotypic vulnerability trait rather than a state effect in recurrent patients. It reveals a possible mechanism of vulnerability in IFN-α-induced depression patients.

The confounding factors did not interfere with the risk of recurrent depression in our study. However, there was a preponderance of women in depression recurrence,^38^ and female sex was found to be an independent predictor of the emergence of major depression during HCV therapy; women more frequently reported depressive symptoms and more frequently initiated antidepressant medication.^24^ In our cohort, female IFN-α-induced depression patients had a lower full remission rate, and only 81 female subjects (51.2%) were enrolled in the cohort (compared with 75 male subjects (60.5%); Table 5). Although male patients had a significantly higher risk for recurrent depression, this increase did not influence the final results. Other confounding variables were anxiety disorder and hypnosis use. In the IFN-α-treated population, patients reporting sleep disturbances had a higher risk of developing major depression.^24^ In our sample, there was a higher proportion of zolpidem users and anxiety disorder patients in the IFN-α-induced depression group, but neither psychiatric factor interfered with the results of recurrent depression risk (Table 4).

Major depressive disorder is a highly recurrent illness, and prior episodes were significant predictors of a recurrence.^1^ The risk of the recurrence of major depressive disorder progressively increased with each successive episode.^39^ A history of major depressive disorder was also a significant predictive factor of IFN-α-induced depression.^10^ In the matched cohort, IFN-α-induced depression patients with a depressive history might have had a higher risk of recurrence, but the difference did not reach a statistical significance. IFN-α-induced depression could be considered a successive depressive episode and increase the risk of later depressive disorder.

The risk of recurrent depression of the IFN-α-induced depression did not differ from the normal depression group significantly. Because IFN-α-induced depression might have the similar trajectory of normal depression, we further selected the new-onset depressive patients who did not have any IFN-α exposure (*n*=23 886) from the HCV cohort. We compared the IFN-α-induced depression and the normal depression groups for the risk of recurrent depression after the first depressive episode with 1:10 matched randomly by sex and age. The crude and the adjusted HRs of IFN-α-induced depression were 0.84 (0.60–1.18) and 0.82 (0.58–1.16), respectively, compared with the depression group. There was no difference in the risks of recurrent depression between these two groups. The results might support that IFN-α-induced depression was considered as an episode of depressive disorder.

Because those with IFN-α-induced depressive disorder had a higher risk of recurrent depression, the prevention of depressive disorder is important. However, no study has yet addressed a prevention strategy in this specific context, including the optimal duration of treatment of IFN-α-induced depression with antidepressant drugs.^25^ Even in the context of the high rate of IFN-α-induced depression, there is still an ongoing debate on the use of prophylactic antidepressants. In depression, maintenance treatment for several months during remission is essential after an acute episode of depression to prevent relapse, and long-term treatment is necessary to prevent recurrence in patients with more than one episode. Continuation treatment is important in depressive disorders in patients who have responded to initial antidepressant therapy.^40^ For patients who had a depressive episode during IFN-α therapy, the treatment choice was the early discontinuation of IFN-α therapy^41^ and antidepressant therapy.^42^ The long-term management of IFN-α-induced depressive disorder is uncertain,^43^ and further studies are needed to consolidate the potential prevention strategies with drugs and non-drugs for this high-risk group.

### Strengths and limitations

This study had a number of strengths. The participants were drawn from a population-based and highly representative computerized database of medical records spanning 12 years. Because data were obtained from a historical database that collected all available medical information, we can rule out the possibility of recall bias. The pre-IFN-α, IFN-α and post-IFN-α treatment stages were all evaluated. No depression medical records, including antidepressant therapies during the year before IFN-α, were excluded to ensure patients were depression-free and depression was induced by IFN-α. We also excluded depression for at least 6 months after the completion of IFN-α therapy to ensure that IFN-α-induced depression was in complete remission.

The potential limitations of this study should be noted. First, we used only the clinical diagnosis of depressive disorder; no biomarkers were included in the diagnosis. We used outpatient records from three visits or one incident with inpatient records to validate the depression diagnosis. Second, in our study, the patients who needed more than three sessions of therapy were identified as having IFN-α-induced depression. Mildly depressive patients were not identified as our target patients. Most depressive patients who did not need any medical services were considered completely recovered, and we defined depression treatment-naive patients as those without treatment for a year after recovery. IFN-α-induced depression that was not remitted after therapy was not included because this type of depression was thought to be less associated with IFN-α than the depression itself. Third, there were no clinical evaluations of IFN-α-induced depression in our study. We evaluated the early termination of IFN-α therapy and antidepressant therapy during IFN-α-induced depression for the severity of IFN-α-induced depression. Early termination of IFN-α therapy is a possible result of severe depressive symptoms. IFN-α-induced depression can be reversed once treatment has been discontinued.^44^ In our study, IFN-α was terminated within the first 45 days in one-third of subjects, and the risk of recurrent depression was still high. The possible reason for the early termination of IFN-α therapies might be the severity of depression. If IFN-α-induced depression happened and IFN therapy could continue, the depressive symptoms could possibly be tolerated or brought under control. Fourth, several potential unreported confounding factors that are also associated with depressive disorder and IFN-α therapy, including body mass index, family history of depressive disorder, smoking, alcohol intake, over-the-counter drug use and HCV serum levels, were not included in our database. Finally, the study included only Taiwanese patients; the results might not be generalizable to other populations.

## Conclusions

IFN-α-induced depression has a high risk of recurrence that is similar to depressive disorder. It is not a transient disease and might be considered an episode of depressive disorder. Patients who have IFN-α-induced depression should be followed up with carefully not only during the IFN-α therapy but also after the end of therapy. Continuation therapy might be considered, and further research is needed.

## Figures and Tables

**Table 1:** Characteristics of subjects in the interferon-α-treated and matched cohorts

*Variable*	*IFN-α-treated cohort*					*Matched cohort*				
	*IFN-α-induced depression (*n=*156)*		*Without IFN-α-induced depression (*n=*21 452)*		P*-value*	*IFN-α-induced depression (*n=*142)*		*Without IFN-α-induced depression (*n=*1420)*		P*-value*
	*No.*	*%*	*No.*	*%*		*No.*	*%*	*No.*	*%*	
Age (years)					0.066					1.000
<50	49	31.4	6623	30.9		41	28.9	410	28.9	
50–59	69	44.2	7884	36.7		64	45.1	640	45.1	
⩾60	38	24.4	6945	32.4		37	26.0	370	26.0	
										
*Sex*										
Female	81	51.9	9567	44.6	0.67	73	51.4	730	51.4	1.000
										
*Urbanization*					0.58					0.77
Low	13	8.3	1963	9.1		13	9.1	144	10.1	
Moderate	24	15.4	4140	19.3		23	16.2	261	18.4	
High	78	50.0	10 315	48.1		68	47.9	684	48.2	
Very high	41	26.3	5034	23.5		38	26.8	331	23.3	
										
*Income (NTD)*					*0.55*					*0.85*
0	16	10.2	1962	9.2		15	10.6	138	9.7	
1–15 840	24	15.4	2895	13.5		22	15.5	191	13.4	
15 841–25 000	87	55.8	11 622	54.2		77	54.2	779	54.9	
>25 000	29	18.6	4973	23.2		28	19.7	312	22.0	
										
*Medical diseases*										
Diabetes	30	19.2	4914	22.9	0.28	26	18.3	243	24.2	0.12
Hypertension	45	28.9	6986	32.6	0.32	43	30.3	448	31.6	0.76
Hypercholesterolemia	39	25.0	4032	18.8	0.048	36	25.4	290	20.4	0.17
COPD	30	19.2	3898	18.2	0.73	26	18.3	212	14.9	0.29
Heart failure	2	1.3	488	2.3	0.41	2	1.4	31	2.2	0.54
Coronary heart disease	21	13.5	2909	13.6	0.97	19	13.4	182	12.8	0.85
Chronic kidney disease	2	1.3	397	1.9	0.60	2	1.4	21	1.5	0.95
Liver cirrhosis	21	13.5	3951	18.4	0.11	20	14.1	269	18.9	0.16
Anemia	12	7.7	1813	8.5	0.73	11	7.8	129	9.1	0.59
Thyroid disease	8	5.1	1370	6.4	0.52	7	4.9	103	7.3	0.30
Anxiety disorder	40	25.6	3088	14.4	0.0001	35	24.8	208	14.7	0.0017
Zolpidem use	79	50.6	6243	29.1	0.0001	70	49.3	434	30.6	0.0001
										
*Past history*										
Depressive disorder	14	9.0	765	3.6	0.0003	17	12.0	170	12.0	1.0
Antidepressant use	33	21.2	4066	19.0	0.49	28	19.7	280	19.7	1.0
										
*IFN-α therapy*										
Mean day (mean±s.d.)	153.4	66.5	144.1	68.7	0.60	152.7	65.3	148.4	71.5	0.17
Mean dose (mean±s.d.)	10.9	4.6	9.9	5.9	0.0001	10.9	4.6	10.2	6.1	0.0001
Mean follow-up day (mean±s.d.)	692.4	740.4	839.1	827.5	0.065	688.5	752.8	845.2	828.2	0.15
										
*IFN-α-induced depression*										
Onset day of IFN-α-induced depression										
⩽45	58	37.2				50	35.2			
45–90	45	28.8				40	28.2			
>90	53	34.0				52	36.6			
										
* *Post-depression IFN-α doses										
⩽2	52	33.3				45	31.7			
2–5	51	32.7				47	33.1			
>5	53	34.0				50	35.2			
										
With antidepressant therapy										
No	44	28.2				42	29.6			
Yes	112	71.8				100	70.4			

Abbreviations: COPD, chronic obstructive pulmonary disease; IFN, interferon; NTD, New Taiwan dollar.

**Table 2:** Multivariable Cox model measured hazard ratios and 95% confidence intervals for recurrent depressive disorder in the interferon-α-induced depression

	*IFN-α-treated cohort*				*Matched cohort*			
	*HR*	*95% CI*	*Adjusted HR*^a^	*95% CI*	*HR*	*95% CI*	*Adjusted HR*^a^	*95% CI*
IFN-α-induced depression	14.4	10.6–19.4	13.5	9.9–18.3	19.0	10.6–34.3	22.2	11.2–44.2
								
*Past depression history*								
No	14.6	10.6–20.2	13.8	9.9–19.0	17.4	9.4–32.3	20.1	9.7–41.6
Yes	12.9	5.8–28.9	11.8	5.2–26.5	41.7	5.0–348.4	46.1	5.1–418.0
								
*Onset day of IFN-α-induced depression*								
⩽45	15.4	9.6–24.6	15.3	9.5–24.7	13.1	5.7–30.5	16.0	6.2–40.9
45–90	9.8	5.4–17.8	8.9	4.9–16.3	18.2	5.7–58.3	19.9	5.2–76.3
>90	16.9	10.5–27.5	14.4	9.4–25.0	36.2	10.5–124.7	45.5	11.7–177.3
								
*Post-depression IFN-α doses*								
⩽2	13.6	8.4–22.1	13.0	8.0–21.2	33.0	9.6–113.4	40.0	11.4–155.2
2–5	10.5	5.9–18.5	9.6	5.4–17.1	8.5	3.4–21.4	9.7	3.5–27.2
>5	18.9	11.6–30.6	17.5	10.8–28.6	29.7	9.9–88.9	31.0	9.6–100.4
								
*Antidepressant therapy*								
No	15.6	8.8–27.6	15.5	8.7–27.6	13.9	5.2–37.3	16.1	5.2–49.3
Yes	14.0	9.9–19.8	12.9	9.1–18.3	22.3	10.6–47.0	25.8	11.3–58.5

Abbreviations: CI, confidence interval; HR, hazard ratio; IFN, interferon.

a Adjusted for age, sex, income, urbanization, hypertension, diabetes, hypercholesterolemia, chronic obstructive pulmonary disease, liver cirrhosis, coronary heart disease, anemia and thyroid disease.

**Table 3:** Traditional multivariable Cox regression for the association of covariates with recurrent depressive disorder

*Characteristic*	*HR*	*95% CI*	P*-value*
*Age (years)*			
<50	1.0		
50–59	0.92	0.75–1.13	0.42
⩾60	0.73	0.57–0.93	0.010
			
*Sex*			
Female	1.0		
Male	1.30	1.09–1.55	0.0044
			
*Medical diseases*			
Diabetes	0.93	0.76–1.15	0.52
Hypertension	1.14	0.94–1.39	0.19
Hypercholesterolemia	1.11	0.89–1.37	0.36
COPD	1.21	0.98–1.49	0.074
Coronary heart disease	0.99	0.77–1.27	0.93
Liver cirrhosis	0.95	0.77–1.19	0.67
Anemia	0.90	0.66–1.23	0.52
Thyroid disease	0.94	0.68–1.32	0.73
Anxiety disorder	1.87	1.53–2.28	<0.0001
Zolpidem use	2.20	1.84–2.64	<0.0001
			
*Past history*			
Depressive disorder	1.53	1.17–2.00	0.002
Antidepressant use	1.05	0.84–1.30	0.70

Abbreviations: CI, confidence interval, COPD, chronic obstructive pulmonary disease; HR, hazard ratio.

**Table 4:** Hazard ratios of depressive risk in the interferon-α-treated and matched cohort, analyzed by subgroup with Cox proportional hazards regression

	*IFN-α-treated cohort*		*Matched cohort*	
	*Without IFN-α-induced depression*	*IFN-α-induced depression; adjusted HR*^a^ *(95% CI)*	*Without IFN-α-induced depression*	*IFN-α-induced depression; adjusted HR* ^a^ *(95% CI)*
*Models*				
History of anxiety disorder	1.0	12.1 (9.9–16.5)	1.0	22.5 (11.0–45.7)
History of depressive disorder	1.0	12.3 (9.1–16.8)	—	—
History of antidepressant use	1.0	13.4 (9.9–18.2)	—	—
History of zolpidem use	1.0	11.1 (8.1–15.1)	1.0	21.0 (10.4–42.4)

*Subgroup analysis*				
Age, years				
<50	1.0	14.1 (8.0–24.6)	1.0	49.0 (7.7–313.0)
50–59	1.0	13.1 (8.5–20.2)	1.0	56.4 (12.2–260.8)
⩾60	1.0	15.2 (7.4–31.3)	1.0	145.9 (1.9–11425)
				
Sex				
Male	1.0	16.5 (10.4–26.3)	1.0	30.1 (9.2–99.0)
Female	1.0	12.6 (8.3–19.0)	1.0	41.5 (11.7–148.0)
				
Medical diseases				
Diabetes				
Yes	1.0	6.9 (2.8–17.3)	1.0	—
No	1.0	15.3 (11.1–21.2)	1.0	21.0 (12.2–36.1)
Hypertension				
Yes	1.0	21.9 (13.2–36.4)	1.0	—
No	1.0	10.9 (7.40–15.9)	1.0	37.7 (12.1–116.9)
Hypercholesterolemia				
Yes	1.0	12.4 (6.5–23.7)	1.0	—
No	1.0	14.1 (9.9–20.0)	1.0	42.2 (14.1–126.1)
COPD				
Yes	1.0	7.2 (2.9–17.8)	1.0	—
No	1.0	15.6 (11.3–21.6)	1.0	25.9 (11.2–59.9)
Liver cirrhosis				
Yes	1.0	18.4 (8.1–41.5)	1.0	—
No	1.0	12.7 (9.1–17.7)	1.0	27.9 (11.7–66.3)
Anxiety disorder				
Yes	1.0	7.4 (4.1–13.2)	1.0	—
No	1.0	15.0 (10.5–21.6)	1.0	17.5 (7.7–39.8)
Zolpidem therapy				
Yes	1.0	8.1 (5.2–12.4)	1.0	—
No	1.0	17.8 (11.4–27.6)	1.0	38.2 (9.2–158.7)
				
* *Past history				
Depressive disorder				
Yes	1.0	8.0 (3.5–18.1)	1.0	—
No	1.0	14.6 (10.5–20.4)	1.0	17.8 (8.8–35.8)
				
Antidepressant use				
Yes	1.0	13.1 (6.7–25.6)	1.0	212.2 (28.2–1594)
No	1.0	14.3 (10.1–20.2)	1.0	20.9 (9.8–44.4)

Abbreviations: CI, confidence interval; COPD, chronic obstructive pulmonary disease; HR, hazard ratio; IFN, interferon.

^a^Adjusted for age, sex, income, urbanization, hypertension, diabetes, hypercholesterolemia, chronic obstructive pulmonary disease, liver cirrhosis, coronary heart disease, anemia and thyroid disease.

**Table 5:** Characteristics of the groups of interferon-α-induced depression with and without remission

*Variable*	*IFN-α-induced depression*	
	*With remission (*N=*156)*	*Without remission (*N=*126)*
*Age (years)*		
<50	49 (31.4)	40 (31.8)
50–59	69 (44.2)	42 (33.3)
⩾60	38 (24.4)	44 (34.9)
		
*Sex*		
Female	81 (51.9)	77 (61.1)
Male	75 (48.1)	49 (38.9)
		
*Urbanization*		
Low	13 (8.3)	15 (12.4)
Moderate	24 (15.4)	25 (20.7)
High	78 (50.0)	56 (46.3)
Very high	41 (26.3)	25 (20.7)
		
*Income (NTD)*		
0	16 (10.2)	12 (9.5)
1–15 840	24 (15.4)	24 (19.1)
15 841–25 000	87 (55.8)	68 (54.0)
>25 000	29 (18.6)	22 (17.4)
		
*Medical diseases*		
Diabetes	30 (19.2)	19 (15.1)
Hypertension	45 (28.9)	44 (34.9)
Hypercholesterolemia	39 (25.0)	20 (15.9)
COPD	30 (19.2)	24 (19.1)
Heart failure	2 (1.3)	5 (4.0)
Coronary heart disease	21 (13.5)	19 (15.1)
Chronic kidney disease	2 (1.3)	1 (0.8)
Liver cirrhosis	21 (13.5)	16 (12.7)
Anemia	12 (7.7)	12 (9.5)
Thyroid disease	8 (5.1)	8 (6.4)
Anxiety disorder	40 (25.6)	49 (38.9)
Zolpidem use	79 (50.6)	71 (56.4)

Abbreviations: COPD, chronic obstructive pulmonary disease; INF, interferon; NTD, New Taiwan dollar.
